# Impact of COVID-19 on Alzheimer’s Disease Risk: Viewpoint for Research Action

**DOI:** 10.3390/healthcare8030286

**Published:** 2020-08-21

**Authors:** Giulia Abate, Maurizio Memo, Daniela Uberti

**Affiliations:** Department of Molecular and Translational Medicine, University of Brescia, 25123 Brescia, Italy; maurizio.memo@unibs.it (M.M.); daniela.uberti@unibs.it (D.U.)

**Keywords:** SARS-CoV-2 infection, neuroinvasivness, ACE2, Alzheimer’s disease risk

## Abstract

In the middle of the coronavirus disease 19 (COVID-19) outbreak, the main efforts of the scientific community are rightly all focused on identifying efficient pharmacological treatments to cure the acute severe symptoms and developing a reliable vaccine. On the other hand, we cannot exclude that, in Severe Acute Respiratory Syndrome Coronavirus 2 (SARS-CoV-2) positive subjects, the virus infection could have long-term consequences, leading to chronic medical conditions such as dementia and neurodegenerative disease. Considering the age of SARS-CoV-2 infected subjects, the neuroinvasive potential might lead/contribute to the development of neurodegenerative diseases. Here, we analyzed a possible link between SARS-CoV-2 infection and Alzheimer’s disease risk, hypothesizing possible mechanisms at the base of disease development. This reflection raises the need to start to experimentally investigating today the mechanistic link between Alzheimer’s disease (AD) and COVID-19 to be ready tomorrow.

## 1. Introduction

Dementia is one of the major causes of disability and dependency among older people worldwide. It can be overwhelming for not only people affected but also their caregivers and families, with a very high-pressure on the direct and indirect healthcare cost. The Global Dementia Observatory has estimated that worldwide around 50 million people have dementia, with nearly 10 million new cases every year. The total number of people with dementia is projected to reach 82 million in 2030 and 152 in 2050 [1,2]. More recently, the World Health Organization (WHO) has recognized dementia as a public health priority. In May 2017, the World Health Assembly endorsed the *Global Action Plan on the Public Health Response to Dementia 2017–2025*, to provide a comprehensive blueprint for action. Several speakers among policy and public health organisms, from different areas including diagnosis, treatment and care, support for caregivers, and research and innovation were involved [3]. Among the different types of dementia, Alzheimer’s disease (AD) is the most common form and may contribute to 60–70% of cases [1,4]. After more than 100 years from its first description, Alzheimer’s disease remains a big challenge to manage, in terms of both diagnosis and treatment. Up to now, no effective cure has been found, and the main reason for this lies in the fact that many dark sides of its causes remain to be deciphered. On the other hand, scientific research has reached an important achievement in identifying the earliness with which some pathological events occur. It is now well established that the disease starts at least 10–20 years before the symptoms’ appearance [5,6], with precise molecular changes (i.e., beta-amyloid load and phosphorylated tau) and biological processes (i.e., neuroinflammation, mitochondrial dysfunction, and oxidative stress) [7,8,9,10,11].

## 2. The Virus Brain Infectious Theory of Alzheimer’s Disease

The link between Alzheimer’s and infectious theory raised by evidence of the presence in AD postmortem brain of pathogens, including Chlamydia pneumoniae, Borrelia spirochetes, Helicobacter pylori, and herpes simplex virus (HSV) [12]. A strong association between herpes simplex virus type 1 (HSV1) and AD risk has also been suggested by higher infection rates of central nervous system (CNS) in carriers of the type 4 allele of the apolipoprotein E gene (APOE-ε4), a well-recognized genetic AD risk [13,14], found also able to accelerate the breakdown of the blood–brain barrier by damaging pericytes inside the blood vessels [15].

Wozniak and Itzhaki showed that latent HSV1 in the brain of APOE-ε4 carriers was intermittently reactivated by events such as immunosuppression, peripheral infection, and inflammation, eventually culminating in the development of AD [16]. In addition, in the postmortem brain tissues of more than 1000 patients with Alzheimer’s disease, increased levels of human herpesvirus 6A (HHV-6A) and human herpesvirus 7 (HHV-7) have been found when compared to brain tissues of healthy-aging subjects or those suffering from a different neurodegenerative condition [17]. One of the open questions related to the virus theory of AD, which keeps it far from being widely accepted, is the lack of well-recognized approval for a causative link between viral infection and chronic neurological dysfunction. However, the biology of viruses that infect the brain and the resultant host responses, together with improved diagnostic tools and the lessons learned from animal models, have raised the possibility of viral causation of a number of more subtle neurological conditions. Viruses can damage neurons, causing their dysfunction (i.e., affecting neurotransmitter release) or even death (i.e., lysing the cells, as occurs with cytomegalovirus, or inducing apoptosis, as shown for vesicular stomatitis virus), or indirectly activate the immune response against infected cells and this could initiate a neurodegenerative process [18].

## 3. Brain Infection of SARS-CoV-2 Results in Neurological Manifestations

Beginning in late December 2019, a novel infection caused by the Severe Acute Respiratory Syndrome Coronavirus 2 (SARS-CoV-2), a positive-strand RNA virus, started spreading to over 100 countries [19,20]. On March 2020, the World Health Organization (WHO) declared the outbreak of a pandemic, which has caused more than 700,000 deaths globally. Although initially coronavirus disease 19 (COVID-19) was thought confined to the respiratory tract causing a severe respiratory syndrome, very soon it became clear that the virus could invade other organs, including CNS. In one of the first studies on 214 COVID-19 patients, Mao et al. found that, among the severe patients, 36.4% displayed neurological manifestations including acute cerebrovascular disease and impaired consciousness [21]. In addition, more recently updated literature is coming related to the neurological association with COVID-19 [22,23,24,25].

The mechanism by which SARS-CoV-2 penetrates inside the cells is mediated by the binding with Angiotensin-Converting Enzyme 2 (ACE2), which is a transmembrane protease that cuts Angiotensin I and II into smaller peptides Ang(1-9) and Ang(1-7) [26]. ACE2 is mainly present in airway epithelia, lung parenchyma, vascular endothelia, kidney, small intestine, and brain [27,28].

Although there are no indications yet how SARS-CoV-2 may invade the CNS, some parallels can be found with other coronaviruses (CoVs), as such the Severe Acute Respiratory Syndrome coronavirus (SARS-CoV) and the Middle East Respiratory Syndrome coronavirus (MERS-CoV). Increasing evidence shows that CoVs may first invade peripheral nerve terminals, and then gain access to the CNS via a synapse-connected route [29,30,31]. In addition, experimental studies using transgenic mice further reveal that CoVs can reach the brain via the olfactory nerves when given intranasally [32,33]. Interestingly, in mild to moderate disease cases of COVID-19, patients reported olfactory (85.6%) and gustatory (88.0%) dysfunctions. Importantly, in about 11% of patients, anosmia occurred prior to any other clinical symptoms [34].

Based on the belief that the tissue distribution of host receptors are generally consistent with the tropism of the virus [35], ACE2 expression and its modulation in CNS might aid in dissecting the invasion rate and distribution of SARS-CoV-2 in the brain area. To this purpose, several studies through analyses of RNA-seq libraries recently investigated expression of viral entry-associated genes in single-cell RNA-sequencing data from multiple tissues from healthy human donors [36,37]. They found that the SARS-CoV-2 entry receptor ACE2 is highly expressed in nasal goblet and ciliated cells [37], corroborating the hypothesis that SARS-CoVs might enter the human brain by olfactory nerves.

Although the distribution of ACE2 in the brain is not fully revealed, Doobay et al. demonstrated the presence of ACE2 protein and mRNA in the mouse brain, predominantly in neurons [38]. Using a selective antibody, they found that ACE2 was widespread throughout the brain, present in nuclei involved in the central regulation of cardiovascular function such as the cardio-respiratory neurons of the brainstem, as well as in non-cardiovascular areas such as the motor cortex and raphe [38]. More recently, Chen et al. investigated the ACE2 expression in the human brain by analyzing data from publicly available brain transcriptome databases [39]. According to this spatial distribution analysis, ACE2 was found relatively highly expressed and selective for specific brain areas, such as the substantia nigra and brain ventricles. Further, when considering a cell-type distribution analysis, the expression of ACE2 was found in many neurons and non-neuronal cells, such as astrocytes, oligodendrocytes, and microglial cells, and in human middle temporal gyrus and posterior cingulate cortex. In the prefrontal cortex and hippocampus, ACE2-expressing cells were relatively few [40].

Another potential route for SARS-CoVs may be to enter cerebrospinal fluid (CSF) throughout the choroid plexus of ventricles and spread to the brain. In fact, in the mouse brain, relatively high expression of ACE2 was found in the choroid plexus of lateral ventricles. Recently, the SARS-CoV-2 has also been found by genetic sequencing in CSF sample from a 24-year-old male COVID-19 patient in Japan [41].

## 4. Possible Impact of SARS-CoV-2 Infection on Alzheimer’s Disease Exacerbation

The concern that SARS-CoV-2 infection could silently initiate or accelerate a neurodegenerative process lasting decades before being manifest may immediately recall Alzheimer’s disease, and the estimation of possible new cases we will have to expect in the next 10–15 years. Currently, no evidence suggests a correlation between COVID-19 and AD development. Today, we can only draw different possible hypotheses that must be experimentally proved in the future.

It is well known that beta-amyloid (Aβ) is a key factor in AD pathogenesis [42,43,44]. It is derived from the cleavage of the transmembrane protein, amyloid precursor protein (APP). The link between Aβ and AD has been initially suggested by the discovery in familial cases of mutations related to three genes, namely amyloid precursor protein (APP), presenilin 1, and presenilin 2, leading all to Aβ accumulation. The beta-amyloid theory of AD has been further corroborated by different in vivo experimental models and human clinical trials. Pharmacological research is currently focused mainly on reducing Aβ burden.

Increasing evidence demonstrates that Aβ load is one of the earliest brain AD-related molecular changes, starting even 20 years before the appearance of symptoms. More recently, an intriguing relation between virus infection and beta-amyloid has been proposed. Soscia et al. provided data supporting an in vivo function for Aβ as an antimicrobial peptide (AMP) [45]. The authors showed a significantly higher antimicrobial activity in whole brain homogenates from AD compared with age-matched non-AD samples, and that AMP action correlated with Aβ levels. Consistent with Aβ- mediated activity, the increased antimicrobial action was ablated by immune-depletion of AD brain homogenates with anti-Aβ antibodies. According to these findings, virus transient infection may contribute to initiate or accelerate Aβ accumulation in the brain, leading to AD. In addition, the positive inflammatory response activated by transient virus infection in the CNS could evolve in an aberrant self-perpetuating innate immune response by the persistent accumulation of cerebral Aβ. Neuroinflammation is another early feature of AD.

Considering the tropism of the virus for tissues expressing ACE2, all the conditions that upregulate ACE2 expression, also at brain level, should increase the risk of virus invasion and potentially contribute for activating molecular processes leading to neurodegeneration.

For example, one of the mechanisms of action ascribed to anti-hypertensive drugs targeting the Renin-angiotensin system (RAS) in lowering blood pressure and ameliorating cardiovascular conditions is the activation of ACE2/Ang-(1-7)/Mas axis, even at CNS. In fact, ACE2 pathway has a pivotal role as a neuromodulator of cardiac baroreflex mechanisms, leading to an increased sensitivity of this system [46,47]. In addition, central Ang-(1-7) prevents norepinephrine release [48] and induces depressor responses [49,50,51] in hypertensive rats, increases bradykinin levels [52], potentiates the hypotensive effects of bradykinin [53], and increases vasopressin [54] and nitric oxide (NO) release [55]. On the other hand, ACE2/Ang-(1-7)/Mas axis showed a neuroprotective role in different experimental paradigms of brain injury, including stroke and cognitive impairment models [56,57,58]. ACE2 neuroprotection has been associated with reduced RAS signaling, including reduced ACE1 activity, Ang- II level, and AT1R expression [59,60,61], as well as reduced oxidative stress and neuroinflammation. In addition, Ang-(1–7) has been shown to mediate long-term potential and synaptic plasticity and then improve cognition in rodents following activation of Mas receptor, whereas the protective effects were abolished in Mas KO mice [62,63].

The reduced incidence of AD found in subjects treated with RAS-targeting anti-hypertensive drugs was attributed to the increased expression of ACE2 in the brain [64,65,66,67,68]. In middle-aged (13–14-month-old) symptomatic AD Tg mice, the activation of ACE2/Ang-(1-7)/Mas axis by diminazene aceturate (DIZE) administration, lowered hippocampal Aβ, neuroinflammation, and restored cognition [69]. Therefore, these findings corroborate a beneficial effect of ACE2 overexpression at CNS even in AD when molecular pathological events are established.

On the other hand, the use of RAS-targeting anti-hypotensive drugs might remain a double-edged weapon, triggering long-term AD-related pathological processes when subjects are exposed to brain virus invasion that eventually could trigger neuroinflammation and Aβ load. In addition, as mention above, ACE2 enhancement induced an increase of NO in the brain, which at physiological levels have positive effects on neuromodulation and immunomodulation, but, when it is produced in relatively high quantities, it becomes neurotoxic, participating to AD development [70,71]. Astrocytes, microglia, and blood-derived macrophages are also involved in NO release in response to persistent infection or even to continual deposition of inflammation triggering mediators, which in AD context may be Aβ (both the soluble and the fibrillar forms) [72,73,74,75]. We have to mention that currently no association between the use of these drugs and increased incidence or the worsening of acute respiratory distress syndrome have been found. However, we cannot exclude a priori that they can have long-term effects at CNS level.

The persistence of CoV infections can also induce a neuro-immune response, thus SARS-CoV-2′s possible ability to infect macrophages, microglia, and astrocytes in the CNS is particularly important. A neurotropic virus can be able to activate glial cells and induce a pro-inflammatory state [76]. Interleukin (IL)-6, an important member of the cytokine storm, is positively correlated with the severity of COVID-19 symptoms [77]. Microglia, the resident innate immune cells in the brain, has long been implicated in the pathology of neurodegenerative diseases. Accumulating evidence suggests activated microglia as a chronic source of multiple neurotoxic factors, including pro-inflammatory mediators and reactive oxygen/nitrogen species, driving progressive neuronal damage. Microglia can become chronically activated by either a single stimulus, such as pathogen infection, or multiple stimuli exposures to result in cumulative neuronal loss with time [78]. Further, we can also hypothesize that SARS-CoV-2 infection targeting also the brain can induce activated microglia in the brain, finally causing chronic inflammation and neurodegeneration.

It is well known that APOEε4 is associated with an increased risk of AD in a dose-dependent manner when compared to the more common APOEε3 (APOE3) allele [79,80]. The inheritance of two copies of APOE4 increases the chance of developing AD by 12 times compared to the risk of a person with two copies of APOE3, while one copy of APOE4 increases the chance of AD by three times and lowers the average age of onset to 76 years [79]. Recently, in the UK Biobank Community Cohort (n = 451,367), the ApoE e4e4 (homozygous) genotype was also found associated with an increased risk of severe COVID-19 infection, independent of preexisting dementia, cardiovascular disease, and type-2 diabetes [81]. Although further investigations are surely needed to understand the biological mechanisms linking ApoE genotypes to COVID-19 severity in depth, it is important to highlight some relatively understudied role of APOE4.

New findings demonstrate APOE4 acts beyond its well-known roles in influencing Aβ pathology and lipid homeostasis, since it has a strong influence in neuronal inflammation, potentially spreading pathological proteins through the brain. In human postmortem studies, gliosis was found to be significantly associated with APOE4 carriers when quantified in different brain regions using multiple markers of activation, including CD68, Human Leucocyte Antigen-DR isotype, and CD64 [68,69]. Therefore, the interplay between APOEε4 phenotype and innate immune response in the brain following virus infection, such as astrocytes and microglial cells, should be well investigated, as a driving force that sustains neuroinflammation and leads to AD exacerbation. All of these hypotheses about the theoretical impact of SARS-CoV-2 brain infection on Alzheimer’s disease risk are summarized Table 1.

## 5. Implications for Research

Several research and funding efforts have been put in place to fight AD, including advances in understanding the primary causes of AD and in identifying effective treatments. However, AD research has to face several complications since molecular changes that trigger AD start to accumulate in the brain many years before disease exacerbation. Neurodegeneration, inflammation, and oxidative stress hit the brain sublatently for a long time before symptoms occur, thus a novel damaging insult that is targeted to the brain can further worsen the changing brain. Thus, from a research point of view, it is important not to ignore the possible connection between the new transient infection that has affected 22 million people worldwide and the AD-risk, and furthermore to support investigating the link between ACE-2 and Aβ in SARS-CoV-2 affected patients. A preventive strategy [82,83,84,85,86] that foresee both pharmacological and non-pharmacological treatment will aid in this perspective.

## Figures and Tables

**Table 1:** Theoretical impact of SARS-CoV-2 brain infection on Alzheimer’s disease risk. Currently, no evidence suggests a correlation between COVID-19 and AD development. Here, we summarize different possible hypotheses that need to be experimentally investigated today to be ready tomorrow.

Possible Response to SARS-Cov 2 Virus Brain Invasion Associated with AD Risk	Supporting Literature	AD Molecular Basis
• Beta-amyloid (Aβ) can act as an antimicrobial peptide and in turn, Aβcan start to be overproduced as a protective mechanism	[45]	Beta-amyloid (Aβ)
• APOEε4 is associated with an increased risk of AD.	[13,14,15,16,79,80,81]	APOEε4
• APOEε4 is associated with higher susceptibility to increasing infection rates in CNS.		
• APOEε4 is able to accelerate the breakdown of the blood–brain barrier.		
• APOEε4 was found also associated with an increased risk of severe COVID-19 infection.		
• APOEε4 is associated with increased gliosis and neuroinflammation.	[68,69,72]	Neuroinflammation
• Aberrant innate immune response and neuroinflammation can occur after SARS-CoV 2 infection.		
• Neuroinflammation is an early feature of AD.		
• SARS-CoV 2 entry gene (ACE2) is also expressed in the brain	[39,40,64,65,66,67,68,69]	Virus neuroinvasion hypothesis
• ACE2 upregulation in the brain can be neuroprotective in AD.		
• Organ-specific expression of ACE2 correlates with the vulnerability to infection by SARS-CoV-2.		
• Despite in minor extent compared to other non-neuronal cells, also microglia expressed ACE-2	[39,40]	Microglial activated phenotype
• SARS-CoV-2 infection can induce microglial activation leading to neuronal loss.		
• Microglia activation and ACE2 enhancement induced an increase of NO in the brain.	[70,71,72,73,74,75,76,77,78]	Oxidative stress
• When NO is produced in high quantities, it becomes neurotoxic, participating in AD development.		

AD, Alzheimer’s disease; Aβ, Beta-amyloid; ACE2, Angiotensin-Converting Enzyme 2; NO, nitric oxide.
